# Biochar immobilized plant growth-promoting rhizobacteria enhanced the physicochemical properties, agronomic characters and microbial communities during lettuce seedling

**DOI:** 10.3389/fmicb.2023.1218205

**Published:** 2023-07-05

**Authors:** Ti-Kun Guan, Qiu-Ying Wang, Jia-Shu Li, Hui-Wen Yan, Qing-Jun Chen, Jian Sun, Chao-Jie Liu, Ying-Yan Han, Ya-Jie Zou, Guo-Qing Zhang

**Affiliations:** ^1^Beijing Key Laboratory for Agricultural Application and New Technique, College of Plant Science and Technology, Beijing University of Agriculture, Beijing, China; ^2^College of Horticulture, Xinjiang Agricultural University, Urumqi, China; ^3^Institute of Forestry and Pomology, Beijing Academy of Agriculture and Forestry Sciences, Beijing, China; ^4^Institute of Agricultural Resources and Regional Planning, Chinese Academy of Agricultural Sciences, Beijing, China

**Keywords:** spent mushroom substrate, biochar, plant growth-promoting rhizobacteria, microbial community, lettuce seedling

## Abstract

Spent mushroom substrate (SMS) is the by-products of mushroom production, which is mainly composed of disintegrated lignocellulosic biomass, mushroom mycelia and some minerals. The huge output and the lack of effective utilization methods make SMS becoming a serious environmental problem. In order to improve the application of SMS and SMS derived biochar (SBC), composted SMS (CSMS), SBC, combined plant growth-promoting rhizobacteria (PGPR, *Bacillus subtilis* BUABN-01 and *Arthrobacter pascens* BUAYN-122) and SBC immobilized PGPR (BCP) were applied in the lettuce seedling. Seven substrate treatments were used, including (1) CK, commercial control; (2) T1, CSMS based blank control; (3) T2, T1 with combined PGPR (9:1, v/v); (4) T3, T1 with SBC (19:1, v/v); (5) T4, T1 with SBC (9:1, v/v); (6) T5, T1 with BCP (19:1, v/v); (7) T6, T1 with BCP (9:1, v/v). The physicochemical properties of substrate, agronomic and physicochemical properties of lettuce and rhizospheric bacterial and fungal communities were investigated. The addition of SBC and BCP significantly (*p* < 0.05) improved the total nitrogen and available potassium content. The 5% (v/v) BCP addiction treatment (T5) represented the highest fresh weight of aboveground and underground, leave number, chlorophyll content and leaf anthocyanin content, and the lowest root malondialdehyde content. Moreover, high throughput sequencing revealed that the biochar immobilization enhanced the adaptability of PGPR. The addition of PGPR, SBC and BCP significantly enriched the unique bacterial biomarkers. The co-occurrence network analysis revealed that 5% BCP greatly increased the network complexity of rhizospheric microorganisms and improved the correlations of the two PGPR with other microorganisms. Furthermore, microbial functional prediction indicated that BCP enhanced the nutrient transport of rhizospheric microorganisms. This study showed the BCP can increase the agronomic properties of lettuce and improve the rhizospheric microbial community.

## Introduction

Owing to delicious taste, rich nutrition and medicinal ingredients, edible mushrooms become very popular all over the world. China is the world largest mushroom producer with the annual output of fresh mushroom of 40.6 million tons in 2020 (statistical data from China Edible Fungi Association, CEFA). However, massive by-products, commonly known as spent mushroom substrate (SMS), have been generated along with mushroom harvesting with approximately 5 times of mushroom yield (Leong et al., 2022; Xu S. Y. et al., 2022). Although SMS can be recycled in biogas, composting, animal feeds, etc., it still trails the quantity generated and is often discarded inappropriately (Sewu et al., 2019; Xu S. Y. et al., 2022). Moreover, SMS is mainly composed of disintegrated lignocellulosic biomass, mushroom mycelia and some minerals (e.g., lime and gypsum), which makes SMS abundant in organic matter and a suitable feedstock for biochar (Lou et al., 2017; Leong et al., 2022).

Biochar is the porous carbonaceous product which is thermo-chemically converted from various lignocellulosic feedstocks, including agricultural waste (e.g., straw and corncob), forestry waste (e.g., sawdust and wood chips), and organic and industrial waste (e.g., manure and sludges) (Singh et al., 2022). Biochar derived from different feedstock has showed important applications in waste utilization, organic fertilizers, microbial carriers and pollution management, which is crucial for environmental sustainability (Khan et al., 2021; Uday et al., 2022; Amalina et al., 2023; Bolan et al., 2023). SMS can be carbonized at a various pyrolysis temperature range of 250–800°C and applied in soil amendment and environmental restoration (Lou et al., 2017; Leong et al., 2022). Previous study revealed that SMS biochar represented high pH, C/N and specific surface area, and can mitigate N_2_O emission from soil (Xu X. et al., 2022). SMS biochar from mushroom *Ganoderma lucidum* showed high adsorption efficiency toward heavy metals Pb and Cd (Chang et al., 2020). Moreover, SMS biochar demonstrated high removal efficiencies toward chemical oxygen demand in wastewater (Sewu et al., 2019). It significantly reduced EC and organic matter loss, and increased nutrient elements during the composting process of pig manure with rice straw (Chang et al., 2017). Nowadays, promoting effects of biochar in soil microbial communities and plant growth have been widely concerned (Hossain et al., 2020).

Plant growth-promoting rhizobacteria (PGPR) are a group of beneficial microorganisms which mostly inhabit the rhizosphere. They can promote plant growth, increase plant biomass, maintain soil fertility, and protect plant from phytopathogens (Khatoon et al., 2020; Olenska et al., 2020). Moreover, PGPR may not survive or function when they are inoculated in specific environments, since the exogenous microorganisms might be fiercely competed by native genera (Wang et al., 2021). Therefore, suitable carriers are also important in PGPR applications. Biochar represents large specific surface area, abundant surface functional groups and high porosity, which makes it an excellent carrier for microbial colonization and function (Zhao et al., 2022). Rice-husk derived biochar was reported to be a proper carrier for applications of functional microbes into environmental treatment (Feng et al., 2020). Biochar combined with PGPR strains *Paenibacillus polymyxa* and *Bacillus amyloliquefaciens* can improve nitrogen utilization efficiency and soil microbial community in tomato production, which was better than using biochar or PGPR alone (Wang et al., 2021).

In this study, SMS derived biochar (SBC) was performed as the carrier for combined PGPR strains *Bacillus subtilis* BUABN-01 (BS) and *Arthrobacter pascens* BUAYN-122 (AP). Composted SMS (CSMS), SBC, combined PGPR, and SBC immobilized PGPR (BCP) were applied in the lettuce seedling. Physicochemical properties, agronomic characters and microbial communities were further evaluated. This study may provide a better understanding of the effects of SMS biochar and its immobilized PGPR on plant growth and rhizospheric microbial communities, and also an effective utilization for massive SMS.

## Materials and methods

### Materials

SMS of king oyster mushroom (*Pleurotus eryngii*) was obtained from Chinese Academy of Agricultural Sciences. CSMS was obtained from a mushroom company located in Fangshan District (Beijing, China), which was composted for 20 days using spent *Pleurotus ostreatus* mushroom substrate. PGPR strains *B. subtilis* BUABN-01 (BS) and *A. pascens* BUAYN-122 (AP) were isolated and preserved in the laboratory of edible and medicinal fungi, Beijing University of Agriculture (BUA). They can promote the growth of lettuce (Supplementary Figure S1). Seeds of purple leaf lettuce (*Lactuca sativa* L.) “Beizisheng No. 4” were presented by the vegetable research laboratory, BUA. Commercial seedling substrate (CSS) based on peat was purchased from Dahan Hortitech Co., Ltd. (China) and followed the national agricultural standard of plug seedling substrate of vegetables (NY/T 2118–2012). Vermiculite and coir were purchased from local agricultural materials stores.

### Preparation of SMS biochar and biochar immobilized PGPR

The fresh SMS was pulverized into powder by using a crusher, passed through a 40-mesh sieve, and dried to constant weight under 60°C. The SMS biochar (SBC) was produced by pyrolyzing dried SMS powder at 400°C for 2.5 h using a muffle furnace (Xu X. et al., 2022). The obtained SBC was passed through a 40-mesh sieve and sterilized under 121°C for 0.5 h before immobilization. The two PGPR strains BS and AP (1%, v/v) were, respectively, inoculated into LB liquid medium at 30°C and 150 rpm for 24 h. Fermentation broth was subsequently centrifugated at 3,000 rpm for 15 min. The precipitation was adjusted using sterile water with the bacterial density of 1.0 at 600 nm (Zhao et al., 2022). The two suspensions were mixed with equal volume, followed by another centrifugation and dilution with the final bacterial density of 1.0 at 600 nm. The obtained suspension (combined PGPR) was incubated with the above sterilized SBC (2:1, v/v) in an incubator shaker under 30°C and 150 rpm for 24 h. Finally, the biochar immobilized PGPR (BCP) was recovered by filtration, rinsed with sterile water, dried at 35°C, and stored at 4°C till further studies.

### Seedling and sampling

Lettuce seedling experiments were carried out with seven treatments: (1) CK: CSS served as the commercial control; (2) T1: CSMS based blank control containing CSMS, coir and vermiculite with the volume ratio of 3:1:1; (3) T2: PGPR treatment, T1 with combined PGPR (9:1, v/v); (4) T3: biochar treatment, T1 with 5% SBC (19:1, v/v; or 1% biochar supplementation, w/w); (5) T4: biochar treatment, T1 with SBC (9:1, v/v; or 2% biochar supplementation, w/w); (6) T5: BCP treatment, T1 with BCP (19:1, v/v); (7) T6: BCP treatment, T1 with BCP (9:1, v/v) (Table 1). Each treatment was performed in triplicate using seedling plates (28 holes, 6 cm of upper caliber length and width, 6 cm of height). All plants were grown at 21 ± 1°C, 60% humidity and 14 h photoperiod for 28 days in the intelligent control sunlight greenhouse, BUA (Zied et al., 2021). After seedling, the plants, rhizosphere soil and substrate were, respectively, collected for further laboratory studies (Xu S. Y. et al., 2022).

**Table 1:** Substrate formula used for the seedling experiment.

Treatment	Formula (v/v)
CK	Commercial seedling substrate
T1	CSMS: coir: vermiculite = 3: 1: 1
T2	T1: combined PGPR = 9: 1
T3	T1: SBC = 19: 1
T4	T1: SBC = 9: 1
T5	T1: BCP = 19: 1
T6	T1: BCP = 9: 1

### Physicochemical properties and soil enzyme activities of substrate

The physicochemical properties of substrate after seedling were determined, including pH, electrical conductivity (EC), total organic matter (TOM), total carbon (TC), total nitrogen (TN), total phosphorus (TP), available phosphorus (AP), and available potassium (AK) (Xu S. Y. et al., 2022). Soil enzyme activities of substrate after seedling were determined by using testing kits from Beijing Solarbio Science & Technology Co., Ltd. (China), including sucrase (SC), urease (UE), cellulase (CL), catalase (CAT), acid protease (ACPT), alkaline protease (ALPT), acid phosphatase (ACP), and alkaline phosphatase (ALP) with the catalog number of BC0240, BC0120, BC0150, BC0100, BC0806, BC0880, BC0140, and BC0280, respectively.

### Agronomic and physicochemical properties of lettuce

The plants were collected by gently shaking off the root soil and washing with distilled water. The surface moisture was blotted up by absorbent paper. Agronomic properties of lettuce were determined, including plant height (PH), taproot length (RL), leave number (LN), and fresh weight of aboveground (AGFW) and underground (UGFW). The photosynthetic parameters were evaluated on a sunny morning as described by Xu S. Y. et al. (2022), including net photosynthetic rate (*P*_n_), intercellular CO_2_ concentration (*C*_i_), stomatal conductance (*G*_s_), and transpiration rate (*T*_r_) (Teng et al., 2022). Leaf chlorophyll content (CC) was determined using a hand-held chlorophyll meter (Konica Minolta, Japan). Leaf anthocyanin content (AC) was measured by spectrophotometry as described by Nakata et al. (2013). Root activity (RA) and root malondialdehyde content (MDA) were determined with root tissue by using the plant dehydrogenase assay kit (BC3125, Solarbio, China) and the MDA assay kit (A003-1, Nanjing Jiancheng, China), respectively.

### DNA extraction and Illumina MiSeq sequencing of rhizosphere

The rhizospheric soil was collected by the shaking root method as described by Xu S. Y. et al. (2022). Total soil DNA was extracted using the E.Z.N.A.^®^ soil DNA kit (Omega Bio-tek, Norcross, GA, United States), following the manufacturer’s protocol. DNA quality was evaluated by 1% agarose gel electrophoresis and a NanoDrop spectrophotometer. The V3-V4 region of bacterial 16S rRNA gene and the ITS1 region of fungal ITS gene were amplified in each sample in quadruplicate with primer pairs of 338F-806R and ITS1F-2R, respectively (Xu S. Y. et al., 2022). Subsequently, the PCR products were recovered, purified, and performed by high throughput sequencing on the MiSeq PE300 platform (Illumina, United States) following the manufacturer’s protocols of Majorbio Bio-Pharm Technology Co., Ltd. (China).

### Bioinformatics analyses

The obtained raw sequence data were quality-filtered and assembled by fastp v0.20.0 and FLASH v1.2.7. Operational taxonomic units (OTUs) were clustered by UPARSE v7.1 based on the sequence similarity over 97%, then identified by databases of Silva v138 (for bacteria) and Unite v8.0 (for fungi) with a confidence threshold of 0.7. The basic bioinformatics analyses were carried out on the online Majorbio I-Sanger Cloud Platform.^1^ The alpha (Chao 1 and Shannon) and beta (Partial Least Squares Discriminant Analysis, PLS-DA) diversities were analyzed in mothur v1.30.2 and R software v4.1.3, respectively. The OTU distribution among treatments were performed by the Venn and Upset plot on R software v4.1.3 and TBtools (Chen et al., 2020). The significant differences in relative abundance between treatments were analyzed using Student’s *t*-test (*p* < 0.05). Potential microbial biomarkers were discovered using the linear discriminant analysis (LDA) effect size (LEfSe) method on the online platform^2^ with an alpha value for the factorial Kruskal-Wallis test of 0.05 and an LDA score threshold of more than 3.0 (Yang Y. R. et al., 2022). The Co-occurrence network analyses were conducted based on Spearman correlation matrix using the “hmisc” and “igraph” package in R software v4.1.3. The strong correlations of each pairwise genera (*r* > 0.3, *p* < 0.05) were retained for the visualization of network using Gephi v0.9.2. The Mantel test was performed between bioinformatics parameters and environment parameters via the online R software^3^ (Yang Y. R. et al., 2022). The raw reads of 16S rRNA and ITS genes were submitted to the Sequence Read Archive (SRA) database of NCBI with the accession numbers of PRJNA838550 and PRJNA838833, respectively.

### Statistical analysis

All the data of physicochemical properties were analyzed using Microsoft Excel 2016 and Origin 2020b, and presented as means ± standard deviation (SD). The significance of the different treatments was tested by one-way ANOVA using SPSS (v.25.0). The significance level of differences was set at *p* < 0.05.

## Results and discussion

### Physicochemical properties of substrate

The obtained SMS biochar SBC showed pH of 10.26, EC of 1.00 mS/cm, TN of 1.83%, which were close to biochar derived from spent *P. ostreatus*, *Lentinus* spp. and *Ganoderma lucidum* substrate (Wu et al., 2019; Chang et al., 2020). The physicochemical properties of substrate after the seedling experiment were summarized in Table 2. The CK treatment represented significantly (*p* < 0.05) high levels of EC (1.14 mS/cm) and AK (4.64 g/kg), but significantly (*p* < 0.05) low levels of pH (5.93) and TN (0.53%). Among the SMS based treatments (T1-6), the T4 and T6 treatments (T1 with 10% SBC and T1 with 10% BCP, respectively) demonstrated significantly (*p* < 0.05) high levels of TOM (47.96 and 47.58%), TC (26.65 and 26.44%), TN (1.27 and 1.26%), TP (4.13 and 4.12 g/kg) and AP (1.47 and 1.39 g/kg). Since biochar generally generated from organic waste, it can supply many nutrients such as carbon, nitrogen, phosphorus and potassium (Hossain et al., 2020). Moreover, the TN, TP, AP and AK in the BCP treatments T5 and T6 containing 5 and 10% BCP were significantly (*p* < 0.05) higher than those in the treatments T1 and T2, which was the CSMS blank control and 10% PGPR treatments, respectively. It suggested that the promotion effect of biochar immobilized PGPR was better than that of using PGPR alone. Moreover, there was no significant (*p* < 0.05) difference in pH, TOM, TC, TN, AP, and AK between the two BCP treatments T5 and T6. Many previous studies have also showed that application of biochar can significantly increase soil pH since biochar is alkaline (Singh et al., 2022; Wan et al., 2022). Fang et al. (2020) reported that 2–5% *Eupatorium adenophorum* biochar amendments can significantly improve soil pH by 0.14–0.16 units. Wheat straw biochar application at 20 and 40 t/ha significantly increased soil pH by 2.01–5.43% (Zhang X. et al., 2022). However, 5–10% biochar addition did not significantly (*p* < 0.05) affect the pH value of the SMS based treatments in this study. It might indicate that SMS exhibited an appropriate pH buffering capability.

**Table 2:** The physicochemical properties of substrate after the seedling experiment.

Treatment	pH	EC (mS/cm)	TOM (%)	TC (%)	TN (%)	TP (g/kg)	AP (g/kg)	AK (g/kg)
CK	5.93 ± 0.08 b	1.14 ± 0.16 a	45.99 ± 1.06 ab	25.55 ± 0.59 ab	0.53 ± 0.03 c	2.31 ± 0.16 d	1.18 ± 0.09 b	4.64 ± 0.18 a
T1	8.56 ± 0.20 a	0.88 ± 0.08 bcd	45.00 ± 0.91 bc	25.00 ± 0.51 bc	1.12 ± 0.02 b	3.26 ± 0.05 c	1.12 ± 0.12 b	1.75 ± 0.22 c
T2	8.52 ± 0.09 a	0.97 ± 0.14 abc	42.71 ± 0.39 c	23.73 ± 0.21 c	1.16 ± 0.03 b	3.35 ± 0.11 c	1.23 ± 0.03 ab	1.96 ± 0.05 c
T3	8.70 ± 0.15 a	0.87 ± 0.03 cd	47.13 ± 1.05 ab	26.18 ± 0.59 ab	1.23 ± 0.02 a	3.76 ± 0.11 b	1.32 ± 0.03 ab	2.34 ± 0.10 b
T4	8.86 ± 0.19 a	0.72 ± 0.08 d	47.96 ± 1.77 a	26.65 ± 0.98 a	1.27 ± 0.03 a	4.13 ± 0.08 a	1.47 ± 0.20 a	2.67 ± 0.13 b
T5	8.83 ± 0.27 a	0.82 ± 0.08 cd	46.63 ± 0.78 ab	25.90 ± 0.43 ab	1.27 ± 0.02 a	3.78 ± 0.15 b	1.32 ± 0.01 ab	2.36 ± 0.03 b
T6	8.63 ± 0.17 a	1.11 ± 0.08 ab	47.58 ± 0.31 ab	26.44 ± 0.17 ab	1.26 ± 0.03 a	4.12 ± 0.10 a	1.39 ± 0.03 ab	2.67 ± 0.12 b

The values were expressed as mean ± SD (*n* = 3). Different lowercase letters represent significant differences by Turkey test at *p* < 0.05.

### Soil enzyme activities of substrate

**Figure 1:**
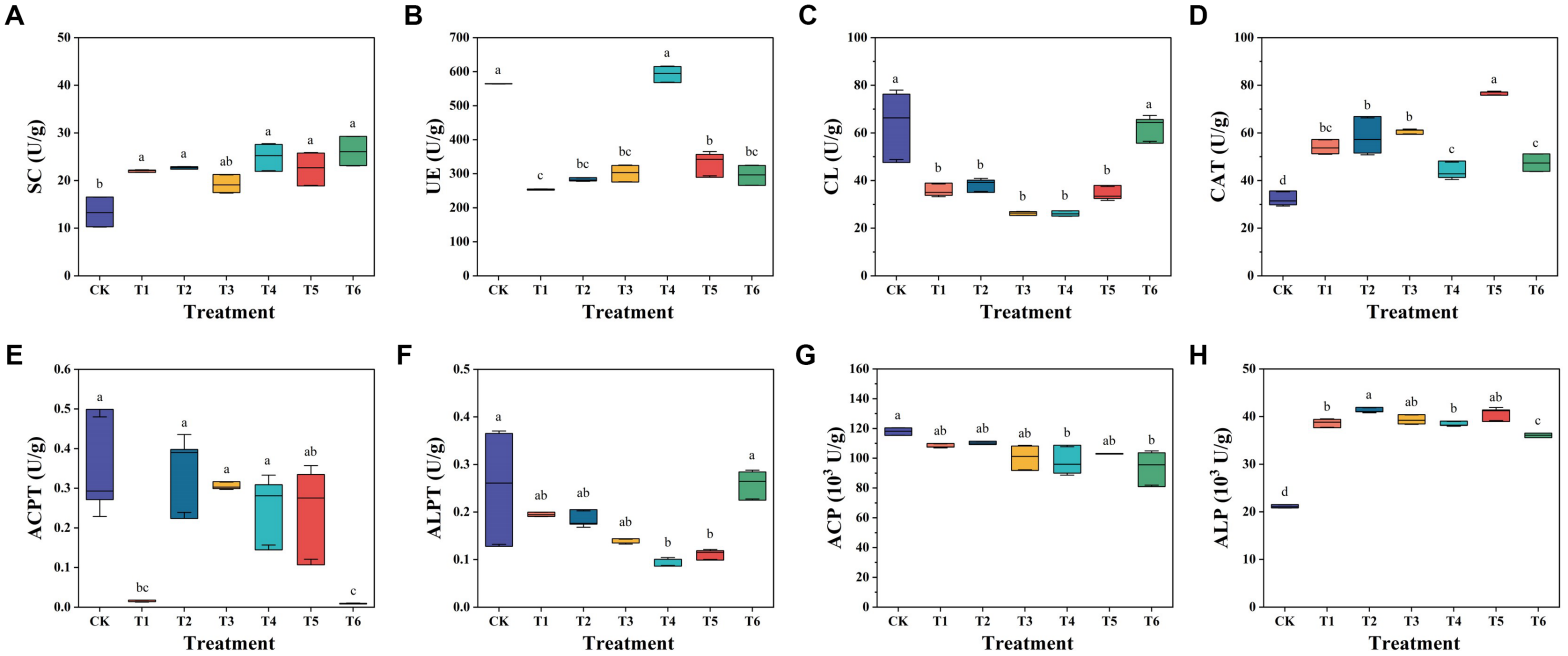
Soil enzyme activities of substrate after the seedling experiment. SC, Sucrase; UE, Urease; CL, Cellulase; CAT, Catalase; ACPT, Acid protease; ALPT, Alkaline protease; ACP, Acid phosphatase; ALP, Alkaline phosphatase. CK, the commercial control; T1, CSMS based blank control; T2, T1 with 10% combined PGPR (v/v); T3, T1 with 5% SBC (v/v); T4, T1 with 10% SBC (v/v); T5, T1 with 5% BCP (v/v); T6, T1 with 10% BCP (v/v). All the data were expressed as mean ± SD (*n* = 3). Different lowercase letters represent significant differences by Turkey test at *p* < 0.05.

To evaluate the impact of SBC and BCP addition, the activities of eight soil enzymes were measured in substrate of all treatments after seedling and as shown in Figure 1. The treatments T1-6 demonstrated significantly (*p* < 0.05) high activities of SC, CAT, and ALP. A previous study also reported that biochar significantly increased soil microbial biomass and UE, ALP, and dehydrogenase activity (Pokharel et al., 2020). In this study, all the six treatments were mainly constituted of composted SMS, which made them had similar high SC activity. They shared an alkaline pH range of 8.52–8.86 (Table 1), which may cause their ALP activities to be higher than that of CK. Moreover, all the six treatments showed a higher activity of ACP than ALP. CATs are antioxidant enzymes that catalyze the conversion of hydrogen peroxide (H_2_O_2_) to water and oxygen, and protect the organisms from oxidative stress (Sooch et al., 2014). The highest CAT activity observed in T5 showed that it demonstrated higher tolerance to oxidative stress. Pheomphun et al. (2019) reported that the increase in *B. cereus* populations was attributed to the significant increase in CAT activity and had the ability to protect *Zamioculcas zamiifolia* against O_3_ stress conditions. It suggested that 5% BCP demonstrated higher protective effects against oxidative stress.

### Agronomic and physicochemical properties of lettuce

**Figure 2:**
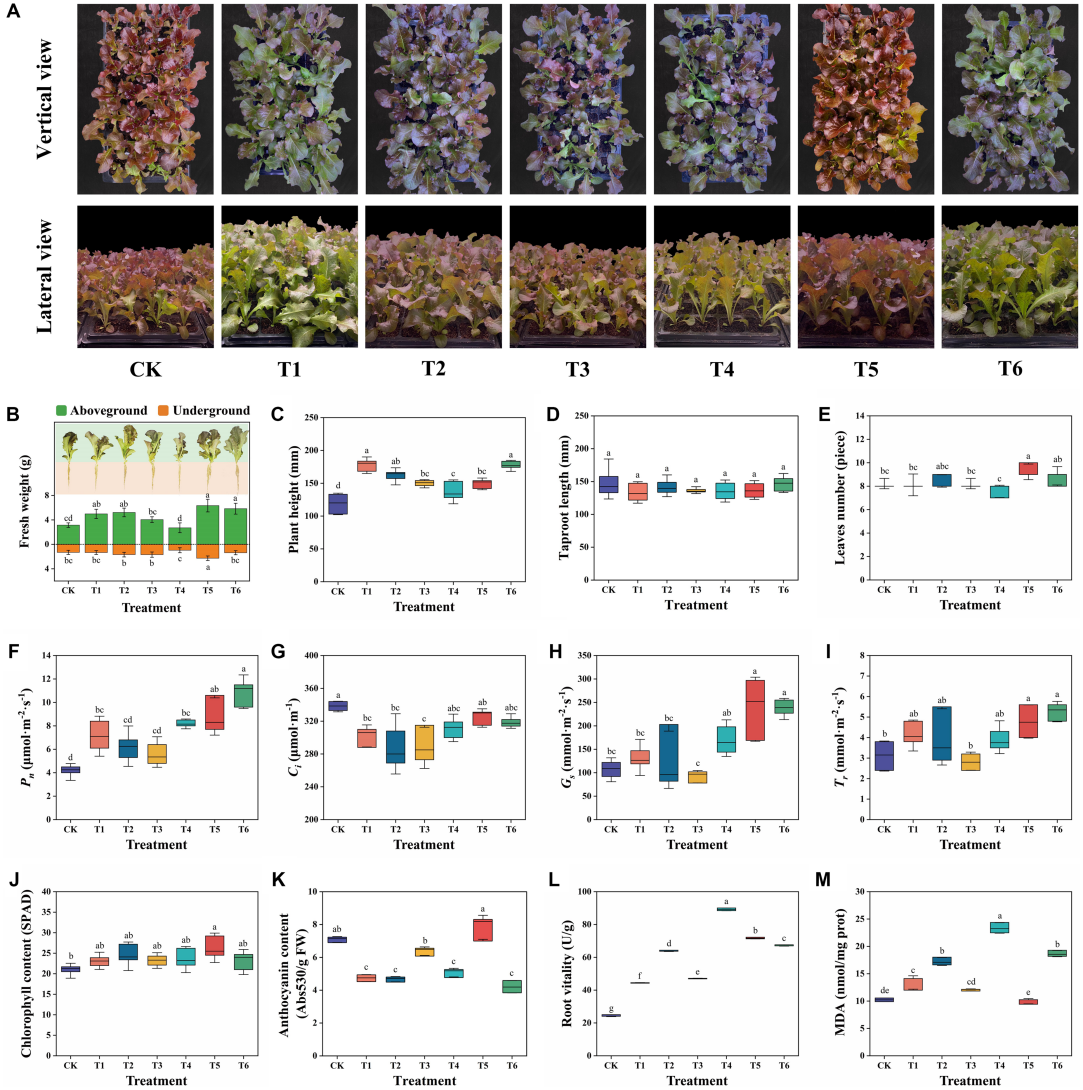
Agronomic and physicochemical properties of lettuce in different treatments. All the data were expressed as mean ± SD. Fresh weight, plant height, root length and leaves number were determined with nine replicates, and the others were determined with six replicates. Different lowercase letters represent significant differences by Turkey test at *p* < 0.05.

After the seedling experiment, agronomic and physicochemical properties of lettuce in different treatments were visualized in Figure 2. The vertical and lateral view of different treatments were showed in Figure 2A. Most of the CSMS treatments except T4 significantly (*p* < 0.05) improved the AGFW and/or UGFW of lettuce relative to the peat based commercial control CK treatment. This indicates that CSMS is a good substitute of the non-renewable peat for seedling cultivation. The T5 treatment demonstrated the highest AGFW (6.22 g), UGFW (2.27 g), and LN (9.22), which were increased by 98.09, 73.28, and 12.17%, respectively comparing with the CK treatment, and 20.54, 69.40, and 13.69%, respectively comparing with the CSMS based blank control T1 treatment (Figures 2B,E). Compared with the T1 and T5 treatments, the T4 treatment containing 10% SBC showed significantly (*p* < 0.05) low agronomic traits, including 2.61 g of AGFW, 0.96 g of UGFW,136.99 mm of PH and 7.56 of LN (Figures 2B,C,E). A previous study suggested that a peat based substrate containing 20% (v/v) wood chip biochar or 30–40% (v/v) rice husk biochar was a favorable growth media for obtaining high-quality *Rhododendron delavayi* seedlings (Bu et al., 2022). Although the recommended dosage of biochar in many previous literatures was 1–5% (w/w) (Fang et al., 2020; Wan et al., 2022), in this study, 10% (v/v) SBC (1% w/w) was not suitable for lettuce seedling. This may be due to the different physicochemical properties of SBC from other biochar.

Moreover, a proper amount of biochar and PGPR can synergistically promote the growth of lettuce. The treatments T5 and T6 with 5–10% BCP both represented high agronomic traits, suggesting that the SBC immobilized PGPR can alleviate the negative effect of high-dose biochar to lettuce. Yang Q. et al. (2022) reported that four types of biochar generally decreased nitrogen and phosphorus content of potato, while the negative effect can be alleviated by arbuscular mycorrhizal fungi (AMF). Wang et al. (2021) also reported biochar had a positive effect on PGPR. When PGPR was used in combination with biochar, the nitrogen use efficiency of tomato was further improved relative to the PGPR without biochar treatment. The combination of biochar and AMF contributed to the whole plant (*Phragmites*) dry weight biomass of 32.30 and 234.00% higher than the single biochar or AMF amendment groups, respectively (Li et al., 2022). Moreover, there was no significant difference (*p* < 0.05) in RL among all the treatment (Figure 2D). The growth promoting tests of the two PGPR strains on the plate showed that AP demonstrated significantly (*p* < 0.05) promoting effects toward AGFW, UGFW, RL and the lateral root number (RN), while BS represented significantly (*p* < 0.05) promoting effects toward AGFW, UGFW and RN, but no significant (*p* < 0.05) effect toward RL (Supplementary Figure S1). These findings indicated that the PGPR (T2), SBC (T3), and BCP (T5) improved the UGFW of lettuce by promoting the growth of lateral roots.

### Photosynthetic and root properties of lettuce

Furthermore, the treatments T5 and T6 demonstrated significantly (*p* < 0.05) high photosynthetic parameters, including *P*_n_, *G*_s_, and *T*_r_, comparing with CK and T1-4 treatments (Figures 2F–I). This suggests that BCP showed better promotion effects on photosynthetic parameters of lettuce than PGPR or SBC used alone. Bu et al. (2022) reported that the appropriate addition of rice husk biochar or wood chip biochar enhanced the photosynthetic activities (*P*_n_, *G*_s_, and *T*_r_) of *R. delavayi* seedling. Huang et al. (2019) declared that the 10% (w/w) straw biochar treatment significantly improved the *P*_n_ of *Phragmites communis* with 64.07% higher than that of the CK treatment. Moreover, the treatment T5 showed the highest chlorophyll content of 26.31 SPAD, which was 26.92% higher than that of the CK treatment. The other CSMS based treatments demonstrated a similar chlorophyll content in a range of 20.73–24.26 SPAD (Figure 2J). It is worth mentioning that the treatment T5 demonstrated the highest leaf anthocyanin content of 7.83 U/g FW with 10.44% and 22.73–85.99% higher than that CK and other CSMS based treatments, respectively (Figure 2K). CSMS (T1) had a significant (*p* < 0.05) negative effect on the leaf anthocyanin content of lettuce with a decreasing rate of 33.15%. Compared with the T1 treatment, PGPR alone (T2) had no significant (*p* < 0.05) effect on the leaf anthocyanin content, while 5–10% SBC (T3 and T4) could significantly (*p* < 0.05) improve the leaf anthocyanin content by 6.97–34.60%. In this study, the tested lettuce “Beizisheng No. 4” was a purple leaf cultivar rich in anthocyanins. After seedling experiment, the leaves in T5 and CK treatments represented bright purple (Figure 1A), which was consistent with the results of the leaf anthocyanin content. Anthocyanins are natural plant pigments with strong antioxidant activity, and play an important role in human health by preventing cardiovascular and neuronal diseases, reducing diabetes and cancer risk, and relieving inflammation (Sandoval-Ramirez et al., 2022). This indicated that 5% BCP could endow lettuce with better nutritional values.

The dehydrogenase activity and MDA content can indicate the root activity and lipid peroxidation level, respectively. The T5 treatment represented significantly (*p* < 0.05) high RA and low MDA content, which was increased by 61.39% and decreased by 25.64%, respectively comparing with the T2 treatment. It also demonstrated an 193.69% higher RA and a 2.5% lower MDA content relative to the CK treatment. Previous studies also reported biochar and PGPR improved the root physiological properties. The combined PGPR *Bacillus subtilis* and *Rhodotorula glutinis* significantly enhanced the RA of lettuce than the blank control (Xu S. Y. et al., 2022). The MDA content in peanut seedling leaves was significantly declined by biochar amendments (Sun et al., 2022). The combination of biochar (30 g/kg) and selenium significantly reduced the MDA content of lettuce (*L. sativa*) by 56% than control (Ma et al., 2022). Moreover, the T2, T4, and T6 treatments demonstrated significantly (*p* < 0.05) high MDA content than the CK and T1 treatments, which indicated that 10% PGPR, 10% SBC and 10% BCP increased the lipid peroxidation level. These findings indicate that the addition of 5% BCP in CSMS is a favorable substrate for lettuce seedling.

### Alpha and beta diversities of microbial communities in seedling substrate

The applications of biochar and PGPR can influence the microbial communities in soil (Singh et al., 2022). DNA-based high-throughput sequencing were performed to evaluate the diversity and succession of microbial communities in different treatments. A total of 40 and 11 phyla, 130 and 34 classes, 347 and 79 orders, 576 and 158 families, 1,113 and 448 genera, and 5,782 and 1,074 OTUs of bacteria and fungi were observed, respectively, with a sequence similarity of >97% (Supplementary Table S1). This suggests that bacteria in the seedling substrate were much more diverse than the fungal species, which is consistent with previous reports (Xu S. Y. et al., 2022; Yang N. et al., 2022). Moreover, Zhang R. et al. (2022) reported that fertilizers had a greater impact on the soil bacterial community than fungal community in a sandy farmland ecosystem.

The alpha diversity can quantitatively reflect the richness and diversity of the microbial communities. The Chao 1 and Shannon indices in different treatments were visualized in Supplementary Figure S2. The T1-6 treatments represented significantly (*p* < 0.05) high bacterial Chao 1 and Shannon indices than CK, while there was no significant (*p* < 0.05) difference among themselves. Moreover, the T1-6 treatments showed significantly (*p* < 0.05) low fungal Shannon index relative to CK, but the fungal Chao 1 index appeared no significant (*p* < 0.05) difference. This indicates that CSMS is more beneficial to promote the bacterial diversity during the lettuce seedling (Xu S. Y. et al., 2022). Moreover, the PLS-DA at the OTU level clustered the bacterial communities in all treatments into five distinct groups, that was, CK, T1, T2, and T3, T4, and T5 and T6 groups (Supplementary Figure S3A). However, the fungal communities into three distinct groups, including CK, T2, and the other treatments (Supplementary Figure S3B). This result validated that SBC, PGPR, and BCP significantly changed the richness and diversity of bacterial communities among different treatments.

The Venn and UpSet plots can be used for reflecting the number and overlap of different OTUs among samples. The results showed that 629 bacterial and 60 fungal OTUs were shared by all the treatments, while 1707 bacterial and 122 fungal OTUs were shared by the CSMS based T1-6 treatments (Supplementary Figure S4). Compared with the T1-6 treatments, the CK treatment had the most unique OTUs (882 of bacteria and 302 fungi). It further suggested that there was a great difference in microbial composition between commercial and CSMS substrate.

### Bacterial community structure in seedling substrate

**Figure 3:**
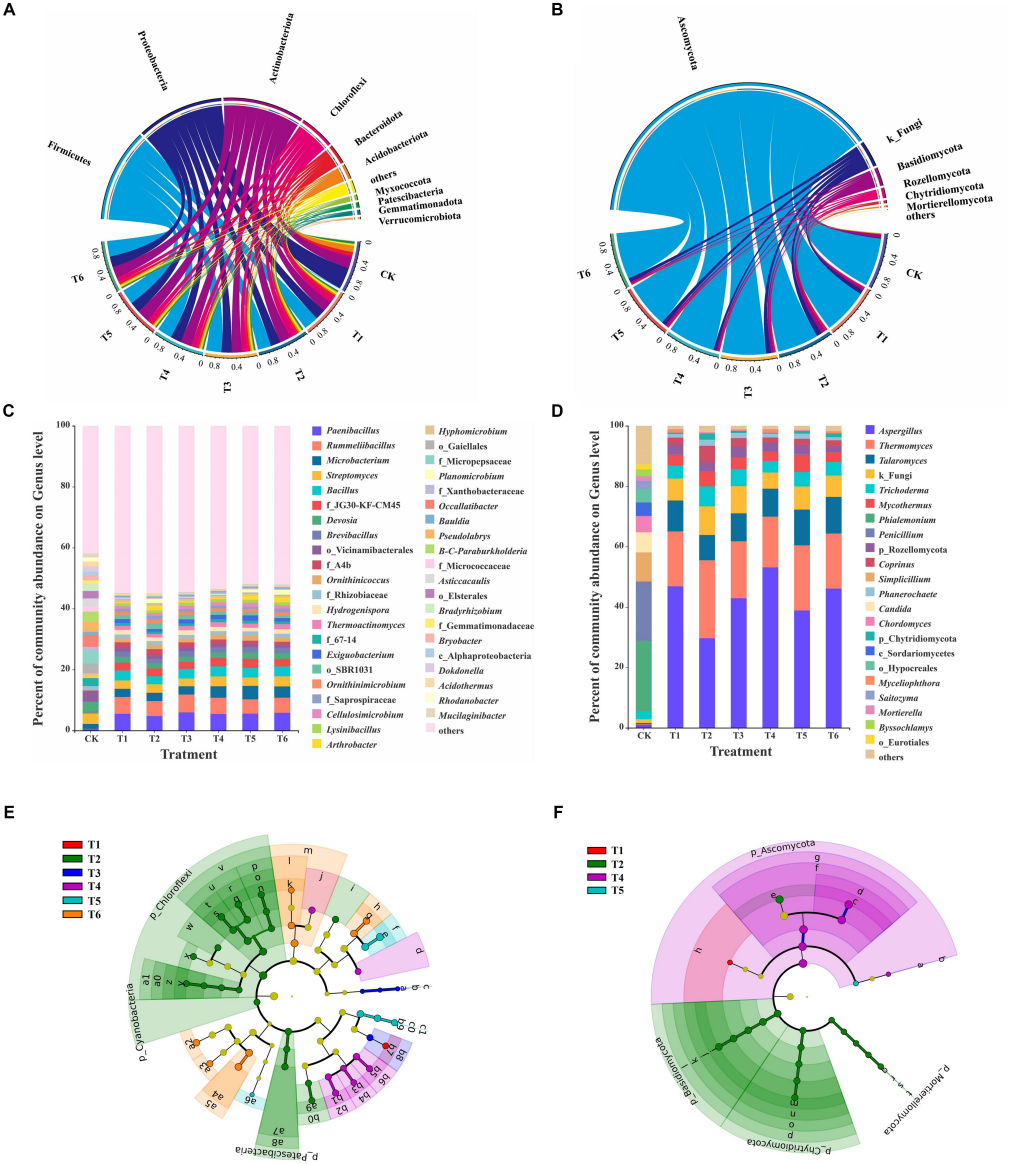
Taxa composition and LEfSe analysis in different treatments. Circos graphs at the phylum level and relative abundance at the genus level of bacteria **(A,C)** and fungi **(B,D)** in different treatments. The relative abundance below 1% were combined together and indicated as “others.” B-C-Paraburkholderia: Burkholderia-Caballeronia-Paraburkholderia. LEfSe analysis identified the significantly different abundant taxa (biomarkers) in different treatments (*p* < 0.05, LDA scores ≥ 3). The circles from the inside to outside indicate phylogenetic levels of bacteria **(E)** and fungi **(F)** from the domain to genus levels. Yellow nodes denote taxa with non-significance, and biomarker taxa are colored by their corresponding class color. (For interpretation of the references to color in this figure legend, the reader is referred to the web version of this article.)

The composition of the bacterial community at phylum and genus levels were shown in Figures 3A–D. Proteobacteria (43.05%) was the dominant phylum in the CK treatment, followed by Actinobacteriota (22.93%), Acidobacteria (13.00%), and Bacteroidota (7.27%) (Figure 3A). The most abundant genera were norank_f__Micropepsaceae (4.17%), *Occallatibacter* (3.90%), *Streptomyces* (3.41%), and Burkholderia-Caballeronia-Paraburkholderia (3.35%) (Figure 3C). Moreover, Firmicutes was the dominant phylum in the T1-6 treatments, where it accounted for 26.87–31.69%, followed by Proteobacteria (20.03–21.41%), Actinobacteriota (20.39–25.00%), and Chloroflexi (8.75–13.04%) (Figure 3A). *Rummeliibacillus* (4.93–5.85%), *Paenibacillus* (4.76–5.84%), *Bacillus* (2.66–3.30%), *Streptomyces* (2.73–3.31%), and *Microbacterium* (2.68–3.69%) were the predominant bacterial genera among T1-6 treatments (Figure 3C). Previous studies reported that Firmicutes, Proteobacteria, Actinobacteria, Bacteroidetes, and Chloroflexi were dominant taxonomic phyla during SMS composting (Hu et al., 2019; Sun et al., 2021). This indicates that the dominant bacterial phyla in the T1-6 treatments mainly came from CSMS. Moreover, a previous study also showed that Proteobacteria (35.9–47.4%), Chloroflexi (8.6–15.8%), Bacteroidota (10.2–14.0%), Actinobacteriota (10.0–11.6%), and Patescibacteria (6.93–11.5%) were the predominant bacterial phyla in the seedling substrate based on the naturally composted SMS (Xu S. Y. et al., 2022). It suggests that different composting methods will affect the dominant bacterial community structure in the composted SMS.

The significant differences analysis in relative abundance between treatments further revealed that a total of 24 genera in the top 25 genera exhibited significant differences (Student’s *t*-test, *p* < 0.05) between CK and T1 treatments (Supplementary Figure S5A). The addition of 10% PGPR significantly (*p* < 0.01) decreased the relative abundance of *Bacillus* from 3.25% (T1) to 2.66% (T2) (Figure 3C and Supplementary Figure S5B). The added *B. subtilis* BUABN-01 was antagonized by local *Bacillus* species, thus reducing the relative abundance of *Bacillus* genus in T2. Although *Bacillus* is one of the most promising bacterial genera for plant growth promotion and has been commercialized, its applications also need to face the stresses of environment and indigenous microorganisms (Sun et al., 2020; Wu et al., 2022). Therefore, carriers such as biochar are combined with PGPR to enhance their promoting effects (Song et al., 2022). There were no significant differences (*p* < 0.05) in the proportion of *Bacillus* between T1 with 5 and 10% BCP (T5-6) treatments (Supplementary Figures S5E,F), suggesting that the biochar immobilization enhanced the adaptability of *B. subtilis* BUABN-01 in the substrate. Moreover, the relative abundance of *Arthrobacter* in T2, T5 and T6 treatments was significantly (*p* < 0.05) higher than that of T1 (Supplementary Figures S5B,E,F). Previous studies reported that *Arthrobacter* spp. increased plant yield and resistance to plant diseases (Schwabe et al., 2021). It suggested that *A. pascens* BUAYN-122 successfully survived in the CSMS based substrate and may promote plant growth.

### Fungal community structure in seedling substrate

The fungal community of the CK treatment at the phylum level was dominated by Ascomycota (90.16%), Basidiomycota (6.64%), and Mortierellomycota (1.52%), whereas Ascomycota (77.38–88.19%), Basidiomycota (3.07–7.67%), and Rozellomycota (2.11–3.55%) were predominant phyla in T1-6 treatments (Figure 3B). Rozellomycota was reported to be one of the predominant phyla in products after SMS composting (Yu et al., 2022), suggested that Rozellomycota in T1-6 treatments came from the CSMS. The CK treatment exhibited a great diversity of fungal community at the genus level relative to that of T1-T6 treatments, with the top five abundant genera of *Phialemonium* (23.23%), *Penicillium* (19.54%), *Simpicillium* (9.64%), *Candida* (6.64%), and *Chordomyces* (5.38%) (Figure 3D). Moreover, the T1-T6 treatments manifested similar dominant fungal composition with the most predominant genera of *Aspergillus* (29.68–46.87%), *Thermomyces* (16.80–25.80%), *Talaromyces* (8.37–12.14%), *Trichoderma* (3.83–6.59%), and *Mycothermus* (3.06–5.74%). These genera are widespread in compost products (Yang Y. R. et al., 2022).

### LEfSe analysis

The LEfSe analysis was further conducted to identify the unique microbial taxa (biomarkers) among T1-6 treatments (Figure 3 and Supplementary Figure S6). A total of 51 bacterial clades significantly enriched among the six treatments, with 1, 23, 4, 8, 6, and 9 biomarkers for T1-6 treatments, respectively (Figure 3E and Supplementary Figure S6A). This indicates that addition of PGPR (T2), SBC (T3-4), and BCP (T5-6) significantly enriched the bacterial diversity in the seedling substrate. *Hydrogenophaga* was significantly enriched in the T1 treatment (CSMS without any addition). The *Hydrogenophaga* species have been isolated from various sources, including compost, water, soil, sludge and mud, and involved in organic degradation (Choi et al., 2020). A total of 17 bacterial biomarkers at the genus level, such as *Arthrobacter*, *Microbacterium, Pedomicrobium*, and *Pseudoxanthomonas*, were significantly enriched in T2-6 treatments, and also reported to be PGPR (Rong et al., 2021). It is worth mentioning that *Arthrobacter* was significantly enriched in T6 treatment, suggesting that biochar enhanced the survival capability of *A. pascens* BUAYN-122 in the substate.

Moreover, there were 24 fungal clades significantly enriched among the six treatments, with 1, 16, 0, 6, 1, and 0 biomarkers for treatments T1-6, respectively (Figure 3F and Supplementary Figure S6B). Fungal biomarkers were less than bacteria among different addition treatments, suggesting that the effects of PGPR, SBC, and BCP addition on bacterial community were higher than that of fungi. The genus *Mortierella* were significantly enriched in the T2 treatment, which was similar with the previous study on microbial inoculation in naturally composted SMS (Xu S. Y. et al., 2022). *Mortierella* is highly abundant in healthy soil, as compared to a high *Fusarium* wilt disease incidence soil (Yuan et al., 2020). It suggested that the combined PGPR improved the microbial community of CSMS based substrate.

### Co-occurrence network of microbial communities

**Figure 4:**
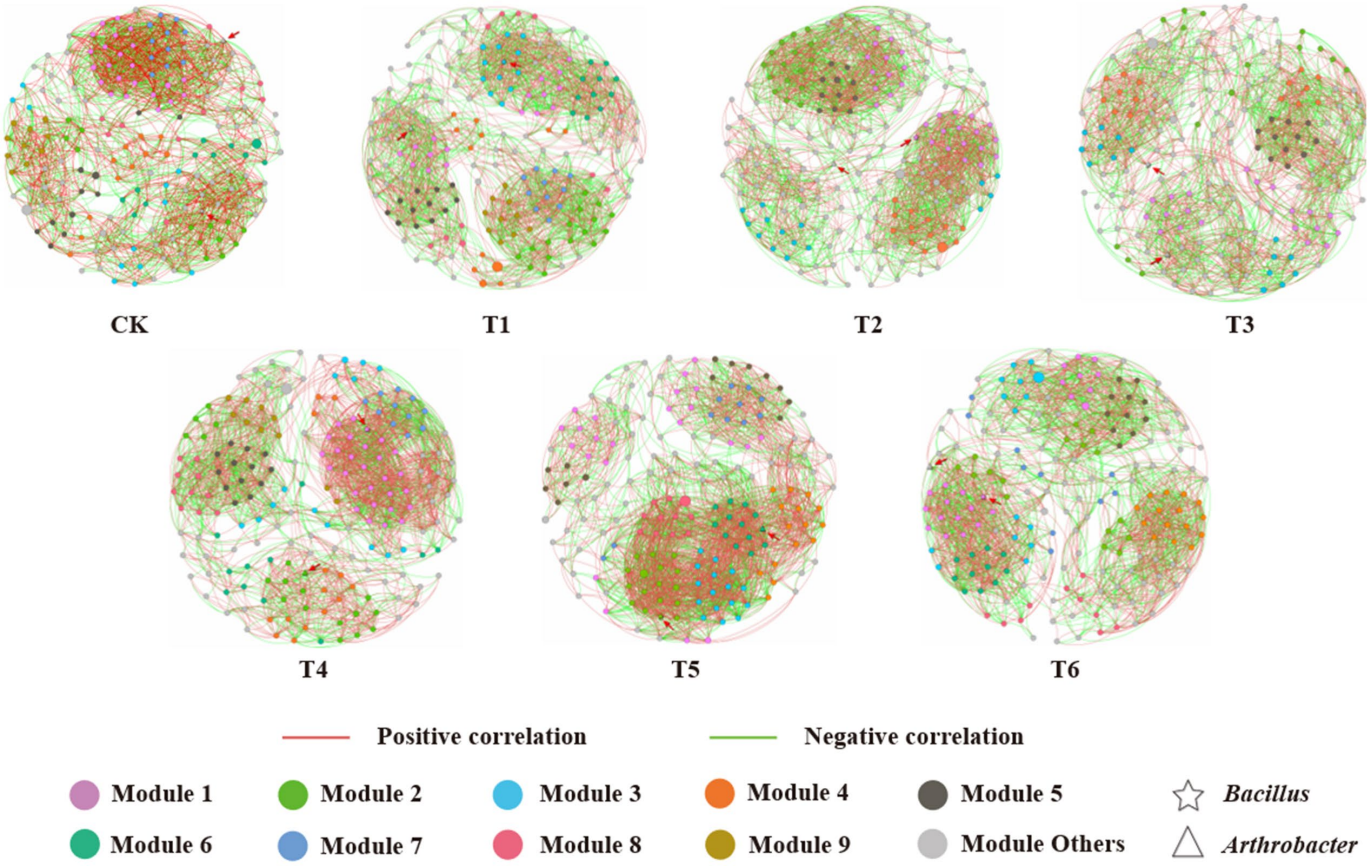
Network model visualizing the co-occurrence patterns of bacterial and fungal communities at the genus level (relative abundance > 0.5%) in different treatments. Each edge represents a significant strong relationship (*r* > 0.6, *p* < 0.05). The red and green edges depict positive and negative correlations, respectively. The nodes represent individual genera, and node size corresponds to their relative abundance. Larger modules with nodes >10 are denoted with different colors, and smaller modules are labeled in gray. (For interpretation of the references to color in this figure legend, the reader is referred to the web version of this article.)

The co-occurrence network analysis was used to explore the complexity of the interactions within the bacterial and fungal communities at the genus level (relative abundance >0.5%) in different treatments (Figure 4). This revealed strong differences in the topological structure of the networks between the CK, T1 and different addition (T2-6) treatments (Supplementary Table S2). A *R*^2^-value range of 0.735–0.829 were registered in the networks of all the treatments, revealing a well-fitted connectivity and scale-free network properties (Bello et al., 2020). The nodes clustering in the co-occurrence network graph indicate that the corresponding microorganisms display functional interdependences and/or occupy similar ecological niches (Mei et al., 2022). Compared to the commercial control (CK), the number of network nodes and edges, and average degree, average path length, average clustering coefficient, network diameter, modularity and percentage of positive links all increased in the CSMS based blank control (T1). Moreover, the application of 10% PGPR further improved the number of nodes and edges, and average degree in the T2 treatment. This indicates that CSMS and PGPR enhanced the relationships in bacterial and fungal communities, which was consistent with previous reports (Zhou et al., 2020; Xu S. Y. et al., 2022). The T5 treatment (5% BCP) demonstrated the highest number of edges and average degree, and the lowest number of nodes, suggesting that BCP increased the network complexity of rhizospheric microorganisms. There were 49 (34 positive and 15 negative) and 56 (16 positive and 40 negative) links of *Bacillus* and *Arthrobacter* with other microbial genera in T5 treatment (Supplementary Table S3). Compared with CK and T1 treatments, 5% BCP greatly improved the correlations of the two PGPR with other microorganisms. This indicates that biochar can provide a suitable habitat for PGPR to play their physiological functions better (Zhao et al., 2022). Moreover, the application of 10% SBC in CSMS (T4) increased the number of edges, average degree and percentage of positive links from 2,389 to 2,498, 25.83 to 27.15 and 51.53 to 60.41%, respectively. Previous studies have shown that the application of biochar can enhance the complexity of microbial co-occurrence network (Mei et al., 2022). Although lettuce in the T4 treatment showed low agronomic characteristics, the addition of biochar still had a positive impact on soil microorganisms.

### Predictive functionality

**Figure 5:**
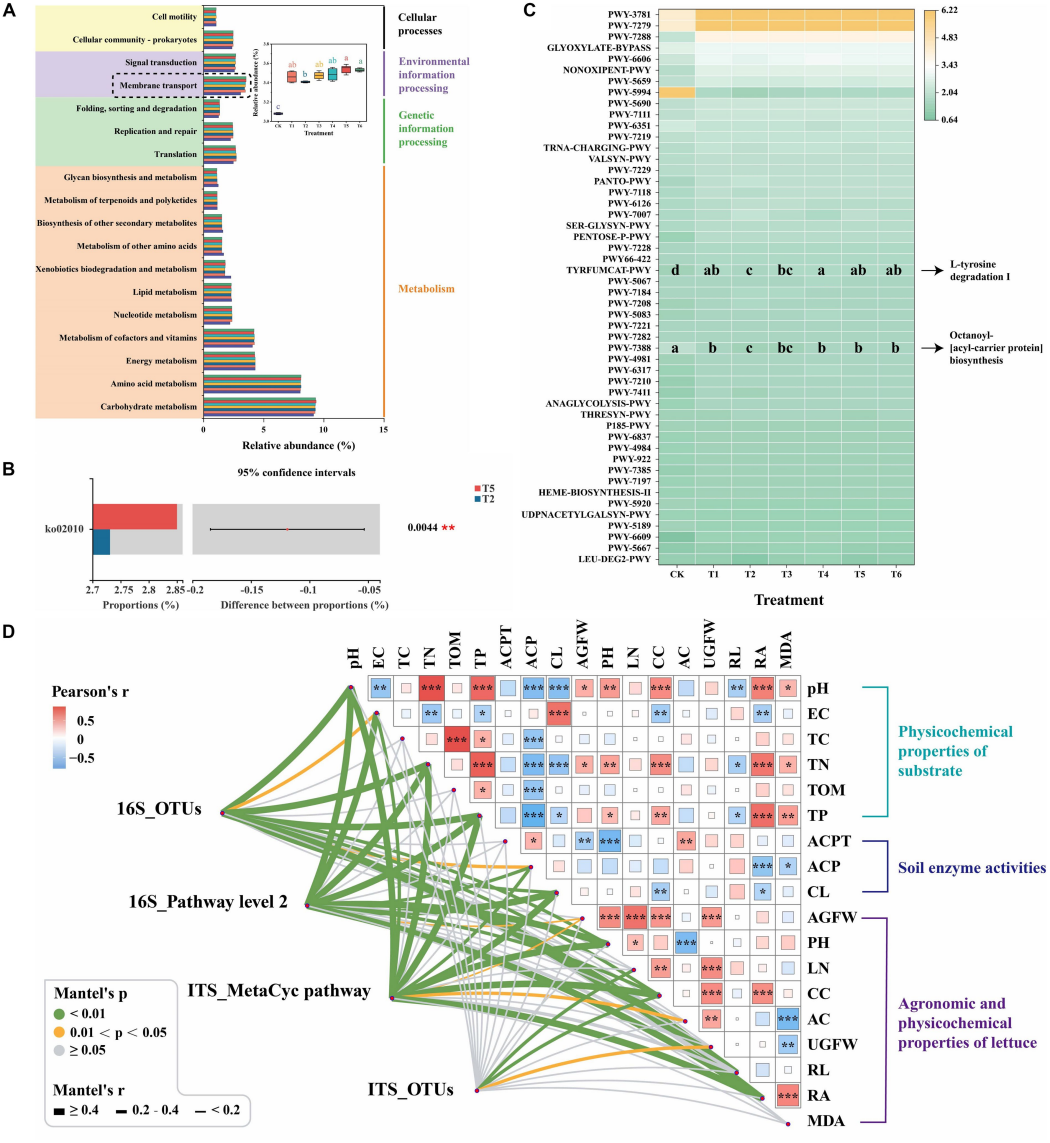
Function predicting and Mantel test analysis. **(A)** The predictive functional features of bacterial sequences mapped against KEGG database (level 2, *p* < 0.05, relative abundance >1%). **(B)** Significantly different relative abundances of bacterial functions between T2 and T5 treatments on the level 3 KEGG ortholog by Student’s *t*-test (*p* < 0.05, relative abundance >1%). **(C)** The predictive functional features of fungal sequences mapped against MetaCyc database (*p* < 0.05, relative abundance >1%). **(D)** Pairwise comparisons between agronomic and physicochemical properties are shown with a color gradient denoting Pearson’s correlation coefficient. The red and green color depict positive and negative correlations, respectively. AC, Leaf anthocyanin content; ACP, Acid phosphatase; ACPT, Acid protease; AGFW, Fresh weight of aboveground; CC, Leaf chlorophyll content; CL, Cellulase; EC, Electrical conductivity; LN, Leave number; MDA, Root malondialdehyde content; PH, Plant height; RA, Root activity; RL, Taproot length; TC, Total carbon; TN, Total nitrogen; TOM, Total organic matter; TP, Total phosphorus; UGFW, Fresh weight of underground. ^*^0.01 ≤ *p* < 0.05, ^**^0.001 ≤ *p* < 0.01, ^***^p < 0.001. (For interpretation of the references to color in this figure legend, the reader is referred to the web version of this article.)

To predict soil bacterial and fungal functions, the Kyoto Encyclopedia of Genes and Genomes (KEGG) and MetaCyc pathway databases were performed using PICRUSt2 (v. 2.2.0) to identify unique pathways and enzymes in different treatments, respectively (Figure 5). Most of the predicted bacterial pathways in different treatments were belonged to four functional groups (pathway level 1): metabolism (77.38–77.73%), genetic information processing (6.06–6.69%), environmental information processing (5.64–6.21%) and cellular processes (4.58–4.67%) (Supplementary Figure S7). Similar observation was reported in the study of combining N fertilization with biochar on rhizospheric soil bacterial communities under sugarcane monocropping (Pang et al., 2022). Moreover, the relative abundance of membrane transport pathway (level 2) of the T5 treatment was significantly (*p* < 0.05) higher than that of the T2 treatment (Figure 5A). This indicates that the application of BCP increased the membrane transport pathway of rhizospheric soil bacteria. The Student’s *t*-test on the level 3 KEGG ortholog between T2 and T5 treatments further revealed that the relative abundance of pathway level 3 ko02010 (ATP-binding cassette transporters, ABC transporters) of the T5 treatment was significantly (*p* < 0.01) higher than that of the T2 treatment (Figure 5B). ABC transporters are primary transporters that can utilize the free chemical energy of ATP to transport a large number of different substances (e.g., essential nutrients) across biological membranes into cells (Thomas and Tampe, 2020). It indicates that BCP may promote the agronomic properties of lettuce by enhancing the nutrient transport of rhizospheric microorganisms. Moreover, the functional prediction of fungal communities based on MetaCyc pathway databases also revealed that the relative abundance of pathways of TYRFUMCAT-PWY (L-tyrosine degradation I) and PWY-7388 (octanoyl-[acyl-carrier protein] biosynthesis) of the T5 treatment was significantly (*p* < 0.05) higher than that of the T2 treatment (Figure 5C). It suggests that the application of BCP improved the nitrogen and lipid metabolism of rhizospheric fungi.

### Correlation analyses

Furthermore, to explore the complex networks of the relationship among four microbial community parameters, six physicochemical properties of substrate, three soil enzyme activities and nine agronomic and physicochemical properties of lettuce were explored by the Mantel test as shown in Figure 5D. The selected physicochemical properties of substrate and soil enzyme activities were based on the heatmap analysis of the Spearman correlation between physicochemical properties and microbial communities (Supplementary Figure S8). The bacterial community (16S_OTUs) significantly (*p* < 0.05) correlated with 10 environmental factors, including ACP, CC, CL, EC, PH, pH, RA, RL, TN, and TP. Meanwhile, the fungal community (ITS_OTUs) represented significant (*p* < 0.05) correlation with three factors of CC, LN and UGFW. This indicates that the bacterial community demonstrated more correlations with the environmental factors during lettuce seedling than the fungal community, which was consistent with the findings of the previous study (Xu S. Y. et al., 2022). Moreover, 16S_Pathway level 2 demonstrated significant (*p* < 0.05) correlation with 10 factors of ACP, AGFW, CC, CL, EC, PH, pH, RA, TN, and TP, whereas ITS_MetaCyc pathway showed significant (*p* < 0.05) correlation with 11 factors of AC, ACP, AGFW, CC, CL, EC, PH, pH, RA, TN, and TP. It is worthy to mention that AGFW of lettuce significantly (*p* < 0.05) correlated with 16S_Pathway level 2 and ITS_MetaCyc pathway, but showed non-significant correlation with bacterial and fungal communities. It suggests that the microbial metabolisms and their products contribute to the plant health. Previous studies reveal the rhizospheric microbial community promote the plant growth and health by secreting various of bioactive compounds, improving nutrition utilization, defensing against pathogens, etc. (Zhalnina et al., 2018; Song et al., 2021). The application of PGPR, SBC and BCP changed the rhizospheric microbiome, which comprised a highly diverse community of microorganisms and can promote plant growth and health.

## Conclusion

In this study, the combined PGPR (*B. subtilis* BUABN-01 and *A. pascens* BUAYN-122), SBC and BCP were used for the promoting effects in lettuce seedling. The results indicated that 5% BCP application in CSMS based substrate demonstrated the best agronomic and physicochemical properties of lettuce and composition of rhizospheric microbial communities. Immobilization of PGPR on SBC enhanced their adaptability in the substrate and increased the network complexity of rhizospheric microbiota. Due to the large output and high nutrient content, SMS is feasible to apply in biochar and its immobilized microbial agents.

## Data availability statement

The original contributions presented in the study are included in the article/Supplementary material, further inquiries can be directed to the corresponding authors.

## Author contributions

T-KG: conceptualization, data curation, software, and investigation. Q-YW, J-SL, and H-WY: data curation and investigation. Q-JC, JS, C-JL, Y-YH, and Y-JZ: funding acquisition, formal analysis, resources, and methodology. G-QZ: conceptualization, data curation, supervision, writing—original draft, and writing—review and editing. All authors contributed to the article and approved the submitted version.

## Funding

This work was financially supported by the Beijing Natural Science Foundation (6232003), Beijing Innovation Consortium of Agriculture Research System (BAIC03-05), and Research Fund for Academic Degree and Graduate Education of Beijing University of Agriculture (2021YJS031).

## Conflict of interest

The authors declare that the research was conducted in the absence of any commercial or financial relationships that could be construed as a potential conflict of interest.

## Publisher’s note

All claims expressed in this article are solely those of the authors and do not necessarily represent those of their affiliated organizations, or those of the publisher, the editors and the reviewers. Any product that may be evaluated in this article, or claim that may be made by its manufacturer, is not guaranteed or endorsed by the publisher.

## Footnotes

^1^www.i-sanger.com

^2^http://huttenhower.sph.harvard.edu/galaxy/

^3^https://www.cloudtutu.com/#/manteltest
