# Supplementary material for: Biochar immobilized plant growth-promoting rhizobacteria enhanced the physicochemical properties, agronomic characters and microbial communities during lettuce seedling
Source: Front Microbiol. 2023 Jul 5;14:1218205. doi: 10.3389/fmicb.2023.1218205 (PMC10354297; doi:10.3389/fmicb.2023.1218205)
Supplement: Supplementary file 1 [file Data_Sheet_1.docx]

**Biochar immobilized plant growth-promoting rhizobacteria enhanced** **the physicochemical properties, agronomic characters and microbial communities during** **lettuce seedling**

Ti-Kun Guan^1^, Qiu-Ying Wang^1, 2^, Jia-Shu Li^1^, Hui-Wen Yan^1^, Qing-Jun Chen^1^, Jian Sun^3^, Chao-Jie Liu^1^, Ying-Yan Han^1^, Ya-Jie Zou^4*^, Guo-Qing Zhang^1*^

^1^ College of Plant Science and Technology, Beijing University of Agriculture, Beijing Key Laboratory for Agricultural Application and New Technique, Beijing 102206, China

^2^ College of Horticulture, Xinjiang Agricultural University, Urumqi 830052, China

^3^ Institute of Forestry and Pomology, Beijing Academy of Agriculture and Forestry Sciences, Beijing 100093, China

^4^ Institute of Agricultural Resources and Regional Planning, Chinese Academy of Agricultural Sciences, Beijing 100081, China

* Corresponding authors.

E-mail: zhanggqbua@163.com (Guo-Qing Zhang)

zouyajie@caas.cn (Ya-Jie Zou)

Tel & Fax: +8610-8079-9143 (Guo-Qing Zhang)

+8610-8210-8681 (Ya-Jie Zou)

**Supplementary caption**

**Supplementary Table A.1.** Taxonomic annotation of bacteria and fungi in different treatments.

**Supplementary Table A.2.** Key topological properties of co-occurring microbial networks in different treatments.

**Supplementary Table A.3.** Network properties of *Bacillus* and *Arthrobacter* with other microbial genera in different treatments.

**Supplementary Figure A.1.** Growth promoting activities of *B. subtilis* (BS) and *A. pascens* (AP) on lettuce seedling. All the data were expressed as mean ± SD (n =3). Different lowercase letters represented significant differences by Turkey test at *P* < 0.05.

**Supplementary Figure A.2.** Microbial richness (Chao 1, a) and diversity (Shannon, b) indices in different treatments.

**Supplementary Figure A.3.** Partial Least Squares Discriminant Analysis (PLS-DA) of bacterial (a) and fungal (b) community at the OTU level.

**Supplementary Figure A.4.** Venn and UpSet plots illustrating bacterial (a) and fungal (b) OTU distribution among treatments.

**Supplementary Figure A.5.** The significant differences in relative abundance of the top 25 genera between treatments by Student’s *t*-test. ^*^0.01 ≤ *P* < 0.05, ^**^0.001 ≤ *P* < 0.01, ^∗∗∗^*P* < 0.001.

**Supplementary Figure S6.** LDA scores of significantly different bacterial (a) and fungal (b) taxa.

**Supplementary Figure A.7.** Bacterial functional predictions of level 1 function categories annotated by PICRUSt2.

**Supplementary Figure A.8.** Heatmap analysis of the Spearman correlation between physicochemical properties of substrate and the top 25 bacterial (a) and fungal (b) genera. ACP: Acid phosphatase; ACPT: Acid protease; ALP: alkaline phosphatase; ALPT: alkaline protease; AK: Available potassium; AP: Available phosphorus; CAT: catalase; CL: Cellulase; EC: Electrical conductivity; TC: Total carbon; TN: Total nitrogen; TOM: Total organic matter; TP: Total phosphorus; SC: sucrase; UE: urease. ^*^0.01 ≤ *P* < 0.05, ^**^0.001 ≤ *P* < 0.01, ^∗∗∗^*P* < 0.001.

**Table A.1.** Taxonomic annotation of bacteria and fungi in different treatments.

|  | Treatment | Phylum | Class | Order | Family | Genus | OTU |
| --- | --- | --- | --- | --- | --- | --- | --- |
| Bacteria | CK | 31 | 91 | 212 | 332 | 562 | 1819 |
|  | T1 | 38 | 125 | 310 | 503 | 935 | 3864 |
|  | T2 | 37 | 117 | 304 | 497 | 913 | 3706 |
|  | T3 | 40 | 124 | 311 | 505 | 932 | 3720 |
|  | T4 | 38 | 122 | 300 | 482 | 897 | 3606 |
|  | T5 | 37 | 117 | 290 | 475 | 885 | 3517 |
|  | T6 | 37 | 117 | 297 | 480 | 892 | 3541 |
|  | Total | 40 | 130 | 347 | 576 | 1113 | 5782 |
| Fungi | CK | 10 | 28 | 65 | 111 | 162 | 473 |
|  | T1 | 8 | 25 | 49 | 83 | 127 | 450 |
|  | T2 | 8 | 22 | 44 | 80 | 131 | 437 |
|  | T3 | 8 | 22 | 35 | 59 | 97 | 372 |
|  | T4 | 7 | 21 | 44 | 78 | 125 | 430 |
|  | T5 | 7 | 21 | 36 | 65 | 103 | 363 |
|  | T6 | 8 | 24 | 45 | 83 | 125 | 399 |
|  | Total | 11 | 34 | 79 | 158 | 448 | 1074 |

**Table A.2.** Key topological properties of co-occurring microbial networks in different treatments.

| Network indices | CK | T1 | T2 | T3 | T4 | T5 | T6 |
| --- | --- | --- | --- | --- | --- | --- | --- |
| Number of nodes | 181 | 185 | 190 | 187 | 184 | 179 | 182 |
| Number of edges | 2192 | 2389 | 2581 | 2177 | 2498 | 2709 | 2312 |
| R square of power law | 0.805 | 0.810 | 0.759 | 0.829 | 0.805 | 0.735 | 0.806 |
| Average degree | 24.22 | 25.83 | 27.17 | 23.28 | 27.15 | 30.27 | 25.41 |
| Average path length | 3.22 | 3.38 | 3.22 | 3.29 | 3.27 | 3.11 | 3.29 |
| Average clustering coefficient | 0.63 | 0.67 | 0.68 | 0.62 | 0.66 | 0.66 | 0.65 |
| Network diameter | 6 | 9 | 6 | 6 | 7 | 7 | 7 |
| Modularity | 0.59 | 0.63 | 0.61 | 0.59 | 0.58 | 0.47 | 0.62 |
| Positive links (%) | 49.36 | 51.53 | 50.45 | 53.19 | 60.41 | 58.80 | 53.20 |
| Negative links (%) | 50.64 | 48.47 | 49.55 | 46.81 | 39.59 | 41.20 | 46.80 |

**Table A.3.** Network properties of *Bacillus* and *Arthrobacter* with other microbial genera in different treatments.

| Treatment | *Bacillus* | | *Arthrobacter* | |
| --- | --- | --- | --- | --- |
|  | Positive links | Negative links | Positive links | Negative links |
| CK | 13 | 16 | 14 | 16 |
| T1 | 19 | 16 | 12 | 18 |
| T2 | 23 | 12 | 6 | 7 |
| T3 | 14 | 4 | 14 | 18 |
| T4 | 11 | 34 | 5 | 18 |
| T5 | 34 | 15 | 16 | 40 |
| T6 | 26 | 11 | 10 | 11 |


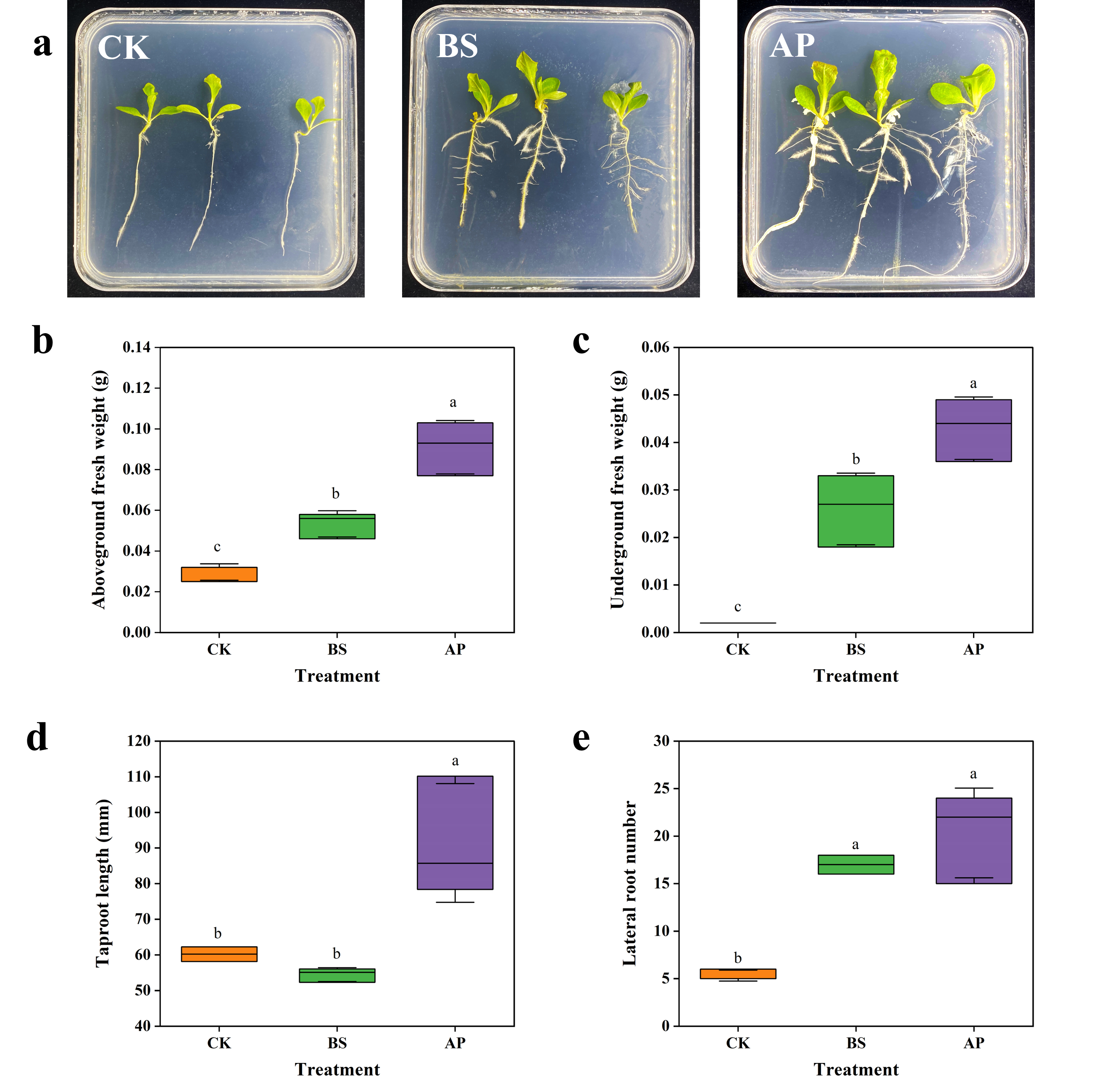


**Fig. A.1.**


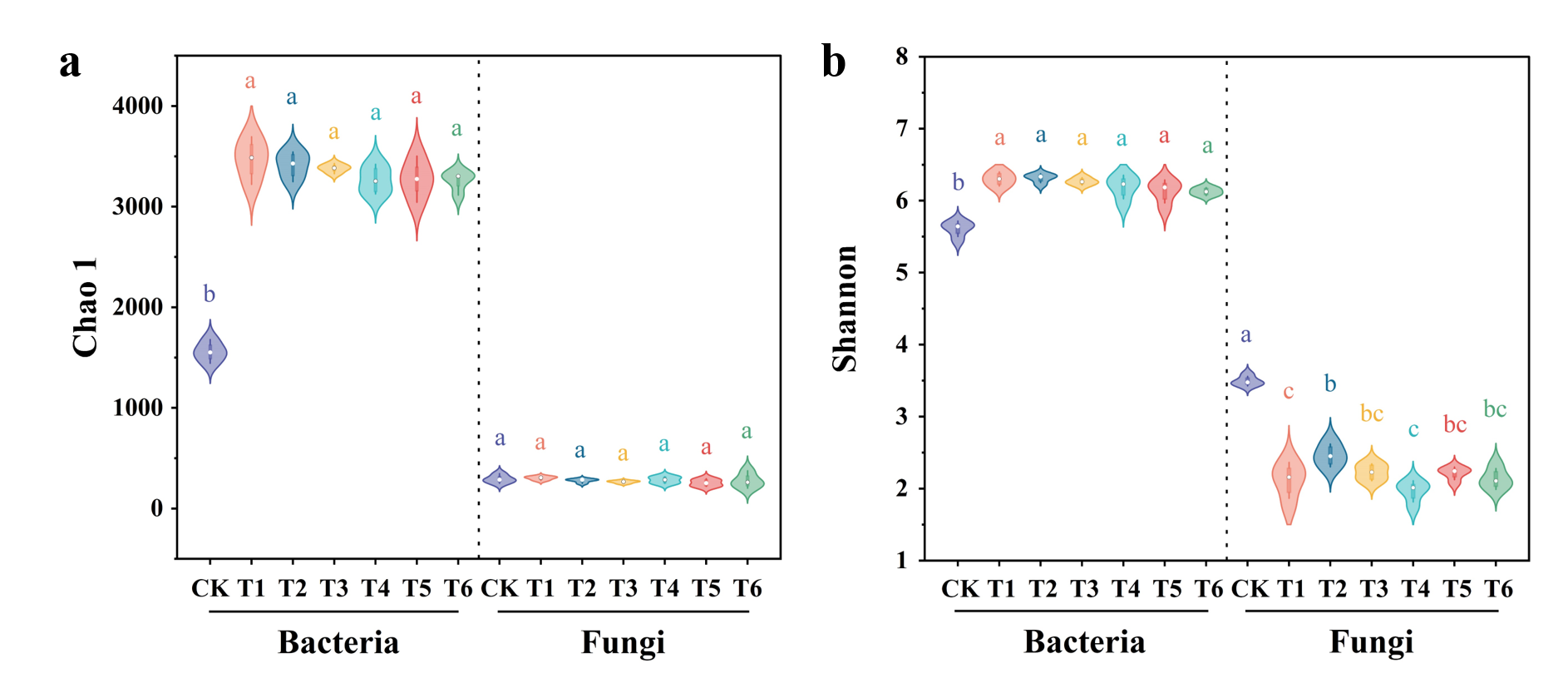


**Fig. A.2.**


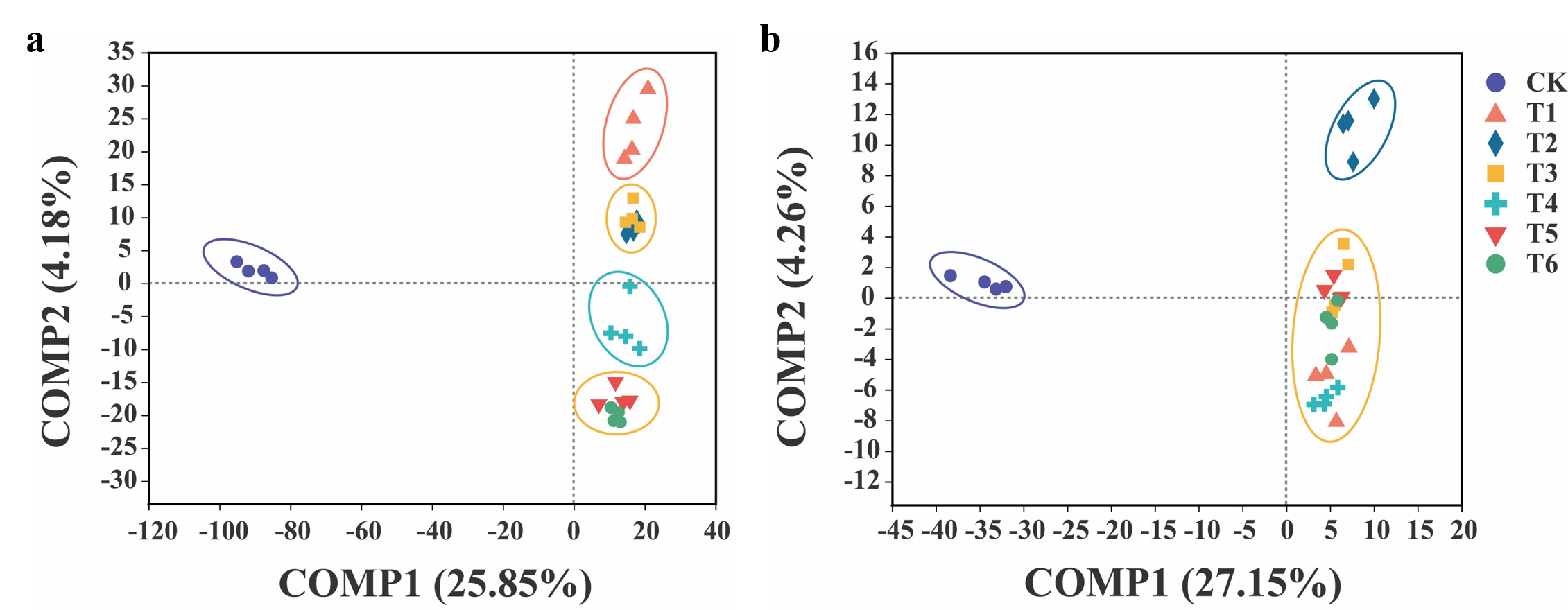


**Fig. A.3.**


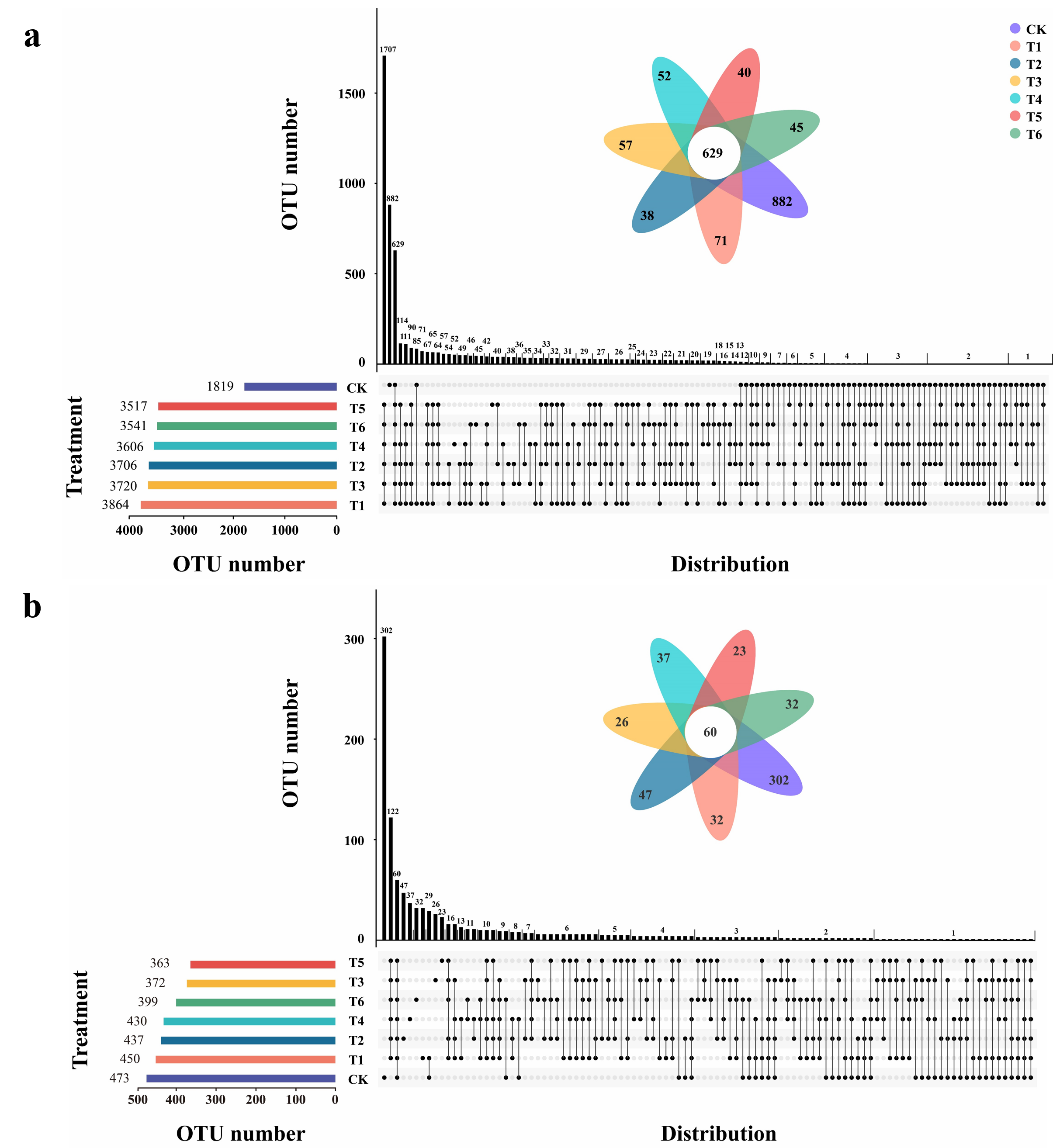


**Fig. A.4.**


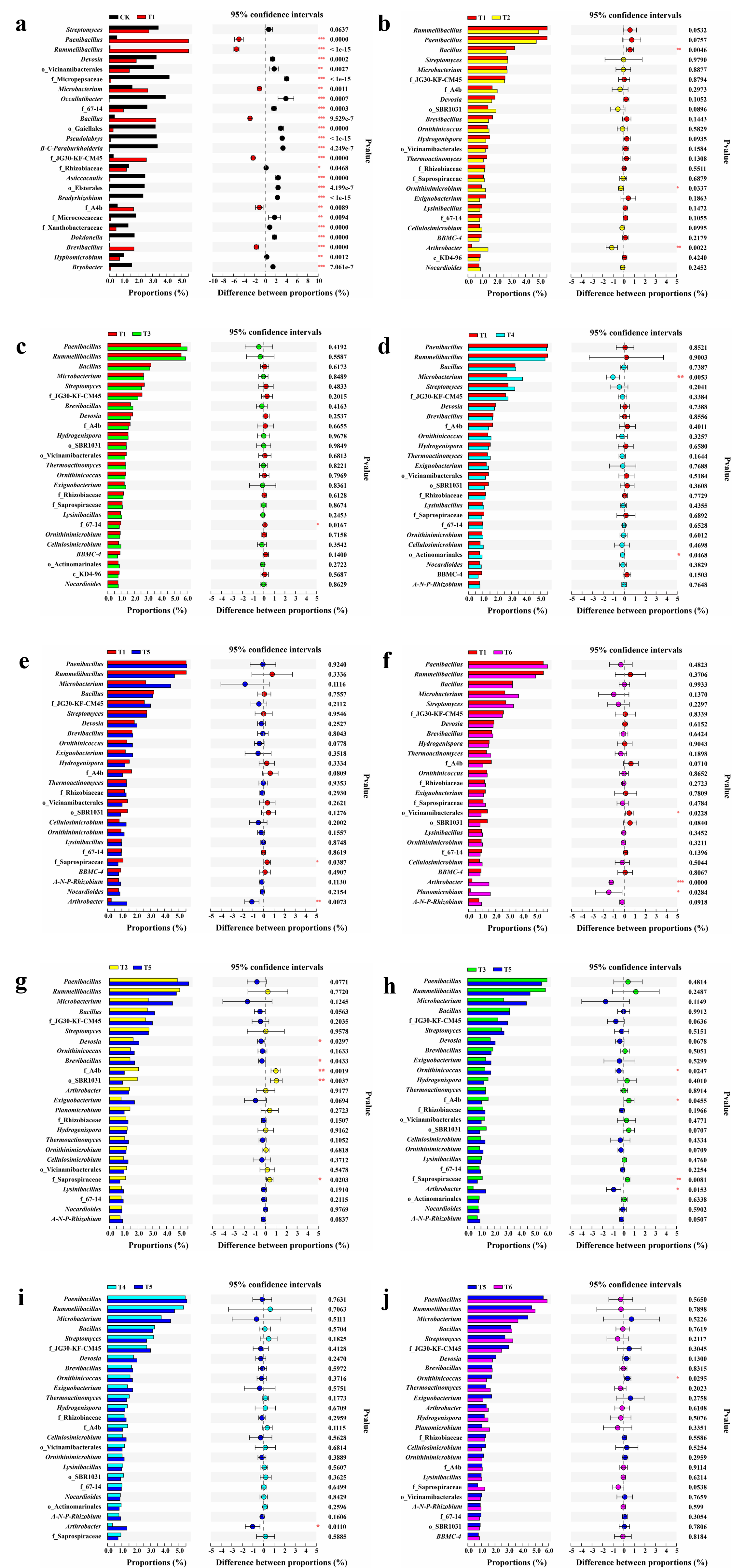


**Fig. A.5.**


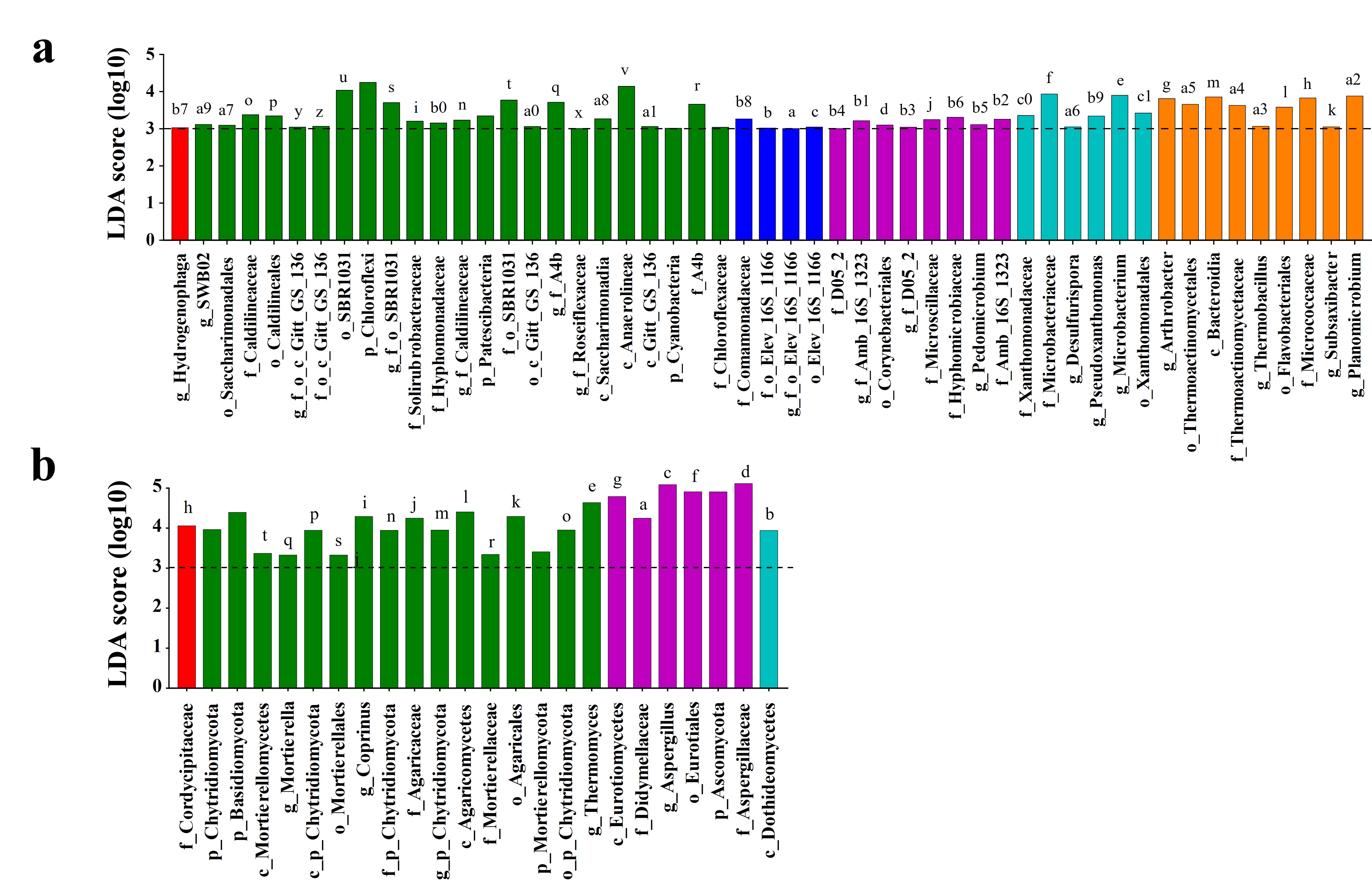


**Fig. A.6.**





**Fig. A.7.**


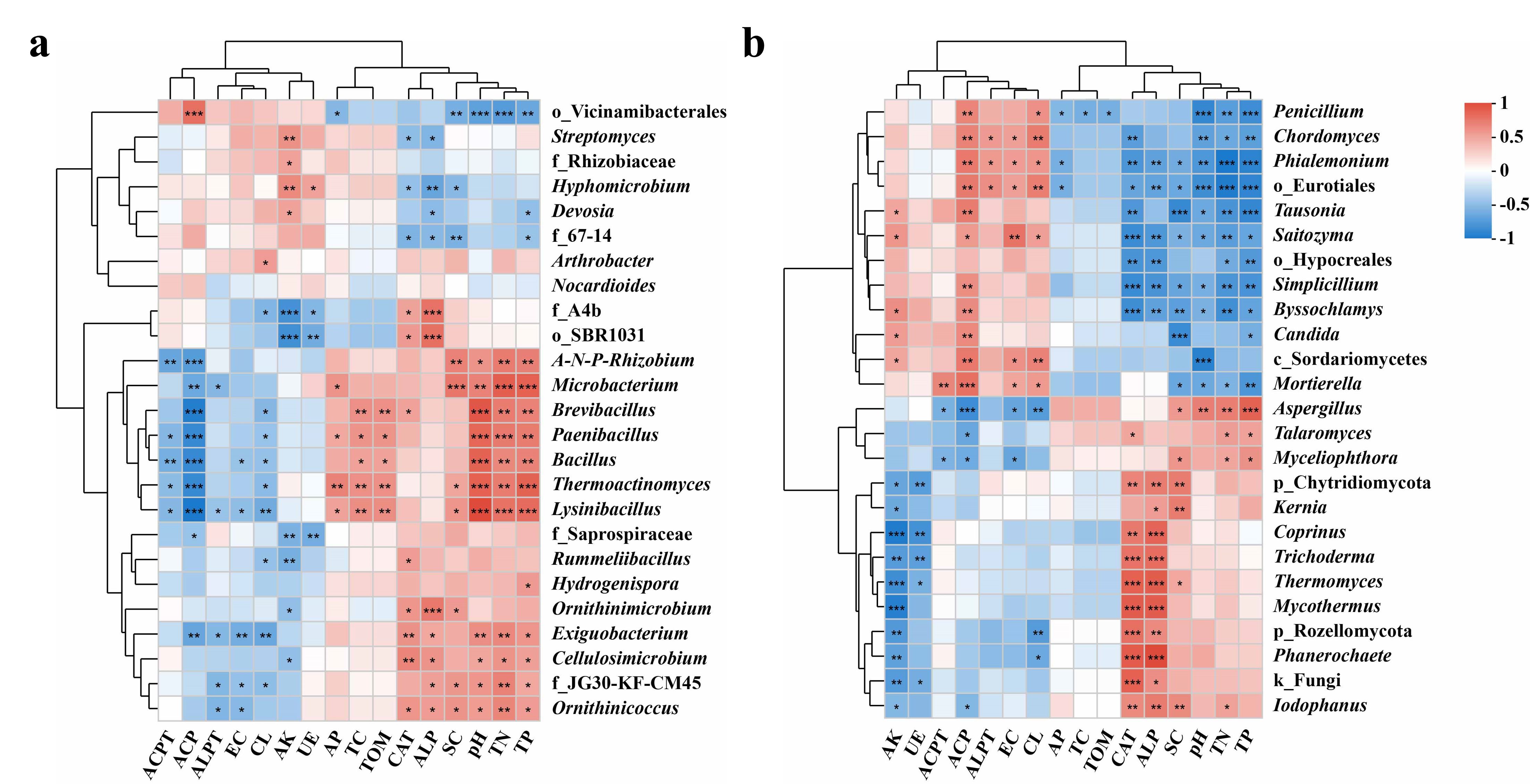


**Fig. A.8.**
